# Flow Diversion Beyond the Brain: Endovascular Management of Wide-Neck Renal Artery Aneurysms Using Flow-Diverting Stents

**DOI:** 10.7759/cureus.91134

**Published:** 2025-08-27

**Authors:** Isabel Carmona, Jochen Gerstner Saucedo, Davood Abdollahian, Orlando Diaz

**Affiliations:** 1 Medicine, Universidad Instituto Colombiano de Estudios Superiores de Incoldo (ICESI), Cali, COL; 2 Diagnostic Radiology, University of Colorado Anschutz Medical Campus, Aurora, USA; 3 Interventional Radiology, Houston Methodist Hospital, Houston, USA; 4 Interventional Neuroradiology, Houston Methodist Neurological Institute, Houston, USA

**Keywords:** endovascular repair, flow diverter stent, hypertension, renal artery aneurysm, vascular surgery, visceral aneurysm, wide-neck aneurysm

## Abstract

The therapeutic management of wide-neck renal artery aneurysms (RAAs) is challenging due to their frequent involvement of bifurcation points and variable shapes, such as lobulated sacs or wide necks that complicate treatment planning. Flow-diverting stents have emerged as an endovascular option for the treatment of complex intracranial aneurysms. However, their application in RAAs remains underreported. In this retrospective case series, we describe three female patients with four wide-neck RAAs treated at a single institution between January 2024 and January 2025. Two aneurysms were located in the left renal artery and two in the right, including one involving a bifurcation. All patients underwent endovascular repair using flow-diverter stents, with or without adjunctive coil embolization. Clinical and imaging data were reviewed. All procedures were successful, achieving complete aneurysm occlusion and preserving renal artery flow. All patients recovered without any complications and were discharged within 24 hours of the procedure. Aneurysm occlusion and preserved parenchymal flow were verified with follow-up imaging at three to six months. Flow-diverters, whether used alone or in conjunction with coil embolization, are a safe and feasible endovascular option for treating wide-neck RAAs, including lesions located at arterial branch points. These findings support the feasibility of minimally invasive treatment in complex renal vascular anatomy.

## Introduction

An aneurysm is a dilation of an artery involving all layers of its wall, resulting in a 1.5 times increase in luminal diameter relative to the normal/adjacent artery [1]. Renal artery aneurysms (RAAs) have been reported in approximately 0.1% of the general population, with an incidence of 0.3% to 2.5% in arteriographic studies; however, their true prevalence remains uncertain [2]. RAAs account for approximately 1% of all aneurysms and 22% of visceral aneurysms [3].

Rundback et al. proposed an angiographic classification, dividing them into three types based on location and morphology: Type I (saccular aneurysms of the main renal artery), Type II (fusiform aneurysms involving bifurcations), and Type III (intralobar branch aneurysms) [4]. Wide-necked aneurysms are a subtype of saccular aneurysm in which the dome-to-neck diameter ratio is less than 1.2, or the neck diameter is greater than 4.0 mm. These are particularly challenging to treat with an endovascular approach due to the risk of coil migration.

In asymptomatic patients with acceptable operative risk, the Society for Vascular Surgery (SVS) clinical practice guidelines on the management of visceral aneurysms recommend treatment of RAAs larger than 3 cm. In women of childbearing age, repair is advised regardless of aneurysm size due to the high maternal and fetal mortality associated with rupture. Other indications for intervention include medically refractory hypertension and functionally significant renal artery stenosis, as multiple studies have demonstrated hypertension resolution following RAA repair [5]. 

Traditional treatment options include open surgical repair and endovascular occlusion. While open repair remains a viable option, endovascular approaches have become increasingly preferred due to their lower perioperative morbidity and mortality and shorter hospital stays [6,7]. However, RAAs often occur at arterial bifurcations or involve branch vessels, complicating both open and endovascular approaches, deeming the procedure technically challenging [8]. Reported complications from open surgery and endovascular approach were 12.4% and 10.5% respectively [9]. However, the endovascular approach had a higher need for reintervention in a longer follow-up period [9]. Because of this, Flow-diverting stents have emerged as a promising alternative for RAAs, offering a minimally invasive solution that preserves renal artery patency while promoting aneurysm thrombosis [6].

Flow diverters were first used to treat cerebral aneurysms, aiding in the treatment of complex aneurysms such as those located at bifurcations or those with wide necks [10]. Recently, their use has expanded to the treatment of complex visceral aneurysms, including RAAs, with multiple individual case reports detailing their use [11].

Despite these advancements, there is limited literature on the application of flow diverters in the treatment of renovascular aneurysms. There are no published case series and only isolated case reports describing the use of flow-diverter stents for the endovascular repair of wide-neck renal artery aneurysms (RAAs), as highlighted in recent systematic reviews and narrative literature reviews, which consistently note the absence of larger series or robust outcome data for this technique in RAAs. This lack of published experience underscores the importance and role of this study in reporting and describing the clinical and radiological outcomes of such interventions, as it addresses a significant gap in the current evidence base and may inform future practice and guideline development. The objective of this study is to report and describe the use of flow-diverter stents for endovascular repair of wide-neck renal artery aneurysms and to evaluate their clinical and radiological outcomes [12,13].

## Case presentation

Study design and patient selection

This is a retrospective, single-center case series conducted at a tertiary care hospital, evaluating the clinical outcomes of patients who underwent endovascular repair of wide-neck RAAs using stents and flow-diverter devices between January 2024 and January 2025. Written Informed consent was obtained for the report of the details of the cases and imaging studies.

Inclusion criteria included a confirmed diagnosis of wide-neck RAA on computed tomography angiography (CTA) and digital subtraction angiography (DSA), treatment with endovascular stent placement or flow diverter (with or without adjunctive coil embolization), and availability of complete preoperative and postoperative clinical data, including follow-up.

Exclusion criteria were patients presenting with ruptured renal aneurysms, a history of prior surgical repair of RAA, or the presence of additional vascular malformations requiring endovascular treatment.

Three patients met all the inclusion criteria, one of whom had bilateral renal artery aneurysms, which required two separate procedures.

Preoperative assessment

Baseline patient characteristics were collected, including demographics (age, sex, race), clinical presentation, comorbidities, and aneurysm size and morphology on the initial imaging study. All images were independently reviewed by two interventional radiologists: the first with over 35 years of experience and the second with eight years of experience, to confirm aneurysm diameter, neck size, and branch involvement. The main characteristics of the included cases are summarized in Table 1.

**Table 1:** Demographic, Clinical, and Imaging Characteristics of Patients Undergoing Endovascular Repair for Renal Artery Aneurysms *All aneurysm measurements reported reflect the maximal measured diameter. FMD: fibromuscular dysplasia; RLQ: right lower quadrant; CTA: computed tomography angiography; HTN: hypertension; AVM: arteriovenous malformation; AVF: arteriovenous fistula; RA: rheumatoid arthritis; GI: gastrointestinal; DM: diabetes mellitus; CTS: carpal tunnel syndrome; DSA: digital subtraction angiography.

Case #	Age	Sex	BMI	Comorbidities & Risk Factors	Presentation	Imaging	Aneurysm Neck Width (mm)	Aneurysm Characteristics *	Radiologic Signs of FMD
One	64	F	28.7	Right adrenal adenoma, prediabetes, hyperlipidemia	RLQ pain → Hematuria	CTA	16	18 mm wide-necked saccular aneurysm involving the bifurcation of the left renal artery	Right renal artery
Two	70	F	29.5	HTN, AVM/AVF, anxiety, hyperlipidemia, osteopenia, RA, prediabetes, visual impairment	Incidental on CTA for suspected GI bleed	CTA	14	16 mm wide-necked saccular aneurysm of the distal right main renal artery	None
Three (a)	53	F	25.4	DM, dyslipidemia, bilateral CTS, restless leg syndrome, family history of aneurysm	Left flank pain	CTA	17	21 mm wide-necked saccular aneurysm in the mid-left renal artery with 60–70% peripheral wall calcification	Left renal artery
Three (b)						DSA	14	14 mm wide-necked saccular aneurysm of the superior division of the right renal artery; 3 mm saccular aneurysm in a right segmental branch	Bilateral (left and right superior segmental arteries)

Procedure technique

Perioperative antiplatelet therapy consisted of aspirin and clopidogrel (Plavix), with platelet inhibition confirmed prior to the procedure using the VerifyNow® P2Y12 assay (Accumetrics, San Diego, CA). Platelet function testing was performed using the VerifyNow P2Y12 assay, which reports results as P2Y12 Reaction Units (PRU) to assess the degree of P2Y12 receptor inhibition. PRU values ranged between 100 and 150, consistent with optimal platelet inhibition. Antibiotic prophylaxis with 2 g of cefazolin was administered. Vascular access was obtained via ultrasound-guided radial or femoral artery puncture.

Case one

A 64-year-old female presented with right lower quadrant pain. Initial CT imaging demonstrated a 17 mm x 15 mm saccular aneurysm with a neck width of 14mm, involving the left renal artery at a bifurcation (Type II). At a later date, the patient developed hematuria, and follow-up CTA demonstrated that the initial aneurysm had grown to 23 mm in maximal diameter and the patient had developed a new 9 mm saccular aneurysm at the level of the left renal hilum.

Via right radial access, a 6 Fr sheath was placed. Via the sheath, a 4 French Glidecath® Cobra 2 catheter (Terumo Medical Corporation, Somerset, NJ, USA) and a Glidewire® (Terumo Medical Corporation, Somerset, NJ, USA) were advanced into the right renal artery. The Glidewire was exchanged for a J-wire, and over this combination, a 6 Fr destination sheath (Terumo Medical Corporation, Somerset, NJ, USA) was advanced into the proximal portion of the right renal artery. The J-wire and base catheter were then removed. DynaCT performed with contrast injection into the right renal artery redemonstrated findings compatible with fibromuscular dysplasia and an 18 mm × 16 mm type II aneurysm of the left renal artery at the origin of the superior pole branch.

Following diagnostic angiography via the left renal artery approach, two Headway 27 microcatheters (MicroVention Inc., Aliso Viejo, CA, USA) were advanced: one into the aneurysm fundus and the other distal to the aneurysm in the superior branch of the left renal artery, which was intended to be preserved. A backstop coil was initially deployed into the aneurysm sac to prevent distal coil migration. Next, a 4.5 mm × 24 mm FRED® flow diverter (Flow Re-Direction Endoluminal Device, MicroVention Inc., Aliso Viejo, CA, USA) was deployed from the superior division to the main renal artery across the aneurysm neck. Through the in-aneurysm microcatheter, multiple MicroVention® coils were then packed into the aneurysm sac, achieving complete occlusion while preserving branch patency.

**Figure 1:**
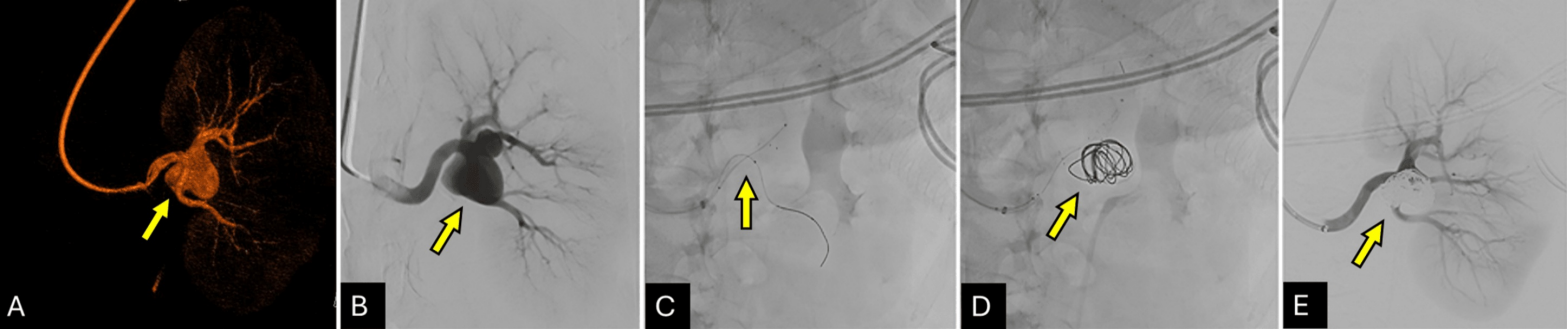
Endovascular Repair of the Left Renal Artery Saccular Aneurysm (A, B) 3D reconstruction angiography and anteroposterior view of subtracted angiography demonstrating the aneurysm at the inferior division of the left renal artery (yellow arrows). (C) FRED® flow diverter stent deployed from the main renal artery into the superior division, anchoring the coil mass (yellow arrow). (D) Coil mass visualized post-deployment (yellow arrow). (E) Subtracted angiography post-embolization showing complete aneurysm exclusion and preserved renal artery patency (yellow arrow).

Final angiography on the left renal artery demonstrated complete occlusion of the aneurysm with preserved flow to the superior pole segmental artery (Figure 1). The patient was discharged the following day without complications. Three-month follow-up imaging confirmed aneurysm stability and vessel patency.

Case two

**Figure 2:**
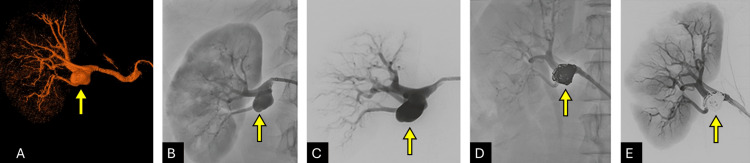
Endovascular Repair of a Right Renal Artery Saccular Aneurysm (A, B) 3D reconstruction angiography and anteroposterior view of subtracted angiography showing aneurysm involving the superior division of the right renal artery (Yellow arrows). (C) Oblique subtracted view of the aneurysm (Yellow arrow). (D, E) Non-subtracted and subtracted images after embolization demonstrate complete aneurysm exclusion and preserved flow through all renal arteries (Yellow arrows).

A 70-year-old female with a past medical history of hypertension presented with symptoms suggesting gastrointestinal bleeding, which prompted an abdominal CTA. Imaging revealed a 15 mm pseudoaneurysm on the distal right main renal artery and a type II wide-necked saccular aneurysm measuring 16 mm × 13 mm, with a neck width of 14mm, extending inferiorly. CTA findings are illustrated in Figure 2.

The procedure was performed under general anesthesia via right common femoral access. A PX Slim microcatheter (Penumbra Inc., Alameda, CA, USA) and a 0.014" Synchro2® guidewire (Stryker Neurovascular, Fremont, CA, USA) were advanced into the aneurysm sac.

Following diagnostic angiography via right common femoral access, a 6 Fr sheath was placed. A 5 Fr Glide Cobra catheter (Terumo, Somerset, NJ, USA) was advanced into the abdominal aorta, and the superior branch of the right renal artery was selected over a Glidewire (Terumo). The Glidewire was then exchanged for a J-wire. Over this wire-catheter combination, a 6 Fr, 45 cm Destination® sheath (Terumo, Somerset, NJ, USA) was advanced into the proximal right renal artery.

To facilitate branch protection and improve control, a Bard Aclipse® 6 mm × 20 mm dual-lumen balloon catheter (Becton Dickinson, Tempe, AZ, USA) was advanced over a 0.014 inch Chikai Black® microwire (Asahi Intecc, Aichi, Japan) into the distal branch of the superior right renal artery supplying the arteriovenous malformation. After balloon inflation, an occlusion arteriogram was performed to delineate the arterial-venous anatomy.

Concurrently, a 2.0 cm PX Slim microcatheter (Penumbra Inc., Alameda, CA, USA) and another Chikai microwire were advanced alongside the balloon catheter and navigated into the venous sac. Balloon occlusion venography confirmed optimal microcatheter positioning. Multiple Ruby® coils (Penumbra Inc., Alameda, CA, USA) ranging from 36 mm to 40 mm were deployed into the venous sac through the PX microcatheter under intermittent arteriographic guidance. A total of six coils were placed.

A repeat angiogram demonstrated markedly reduced flow through the arteriovenous malformation. With the balloon catheter reinflated, a final balloon-occluded arteriogram confirmed diminished filling. Onyx® 34 (Medtronic, Irvine, CA, USA) embolic agent was then administered through the PX microcatheter under balloon occlusion, delivering a total of 0.7 cc, with care taken to prevent off-target embolization.

Final angiography showed a well-expanded device with complete cessation of flow within the aneurysm sac and a preserved perfusion on the inferior pole cortex (Figure 2). The patient tolerated the procedure without complications and was discharged within 24 hours. Follow-up imaging at six months confirmed complete aneurysm exclusion and stent patency.

Case three (a)

A 53-year-old woman with a family history of abdominal aortic aneurysm presented with left flank pain. CTA revealed a wide-necked, type II saccular aneurysm in the midportion of the left renal artery measuring 21 mm × 16 mm × 20 mm, with a neck width of 17mm, and 60-70% of the aneurysm wall showing calcification. Importantly, the inferior branch of the left renal artery originated from the aneurysm neck, which was taken into account during planning and reconstruction.

The procedure was performed under general anesthesia via right common femoral access. Following diagnostic angiography, a 6 Fr sheath was placed. Fluoroscopy confirmed the presence of a 17 mm rim-calcified saccular aneurysm with a wide neck in the midportion of the left renal artery.

To protect distal perfusion and facilitate coil deployment, a 6 mm × 20 mm Eclipse 2L dual-lumen balloon microcatheter (Balt Extrusion, Montmorency, France) was advanced into the inferior division of the left renal artery, crossing the aneurysm neck. Through a secondary PX Slim microcatheter (Penumbra Inc., Alameda, CA, USA) positioned within the aneurysm fundus, multiple Penumbra® and Target® coils (Stryker Neurovascular, Fremont, CA, USA) were deployed under fluoroscopic guidance. Coil packing was performed until complete obliteration of the aneurysm sac was achieved without compromising adjacent branch flow.

To reconstruct the aneurysm neck and maintain downstream perfusion, a 4.5 mm Atlas® stent (Stryker Neurovascular, Kalamazoo, MI, USA) was deployed from the inferior division to the main left renal artery.

**Figure 3:**
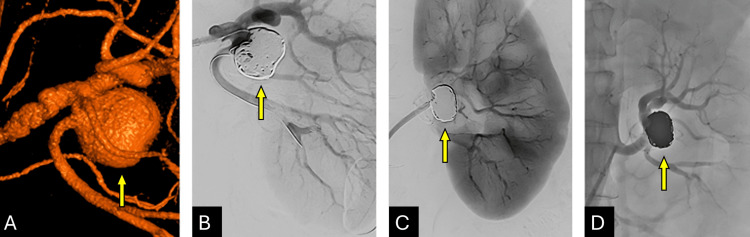
Treatment of a Left Renal Artery Saccular Aneurysm with Calcified Wall (A) 3D reconstruction showing the aneurysm and associated fibromuscular dysplasia (FMD) (yellow arrow). (B) Subtracted image demonstrating the stent deployed from the main renal artery to the inferior division holding the coils (yellow arrow). (C, D) Post-embolization images confirming aneurysm exclusion and preserved renal artery flow (yellow arrows).

Final angiography confirmed complete exclusion of the aneurysm sac, preserved renal perfusion, and no evidence of stenosis, distal embolization, or branch vessel compromise. The Atlas® stent remained patent (Figure 3), confirming technical success. The patient tolerated the procedure well and was transferred to the recovery unit in stable condition.

Case three (b)

During follow-up angiography, two additional aneurysms were identified in the right renal artery of the same patient: a 14 mm wide-necked type III aneurysm in the superior branch with a neck width of 14mm, and a 3 mm aneurysm in a segmental branch. Imaging also demonstrated fibromuscular dysplasia involving the superior division of the right renal artery.

The smaller 3 mm aneurysm in the segmental branch did not meet criteria for endovascular treatment and was not intervened upon during this procedure; it will be monitored with follow-up imaging. The procedure was performed under general anesthesia via right common femoral access. A PX Slim microcatheter (Penumbra Inc., Alameda, CA, USA) and a 0.014" Synchro2® guidewire (Stryker Neurovascular, Fremont, CA, USA) were advanced into the aneurysm sac.

Following diagnostic angiography via right common femoral access, a 6 Fr sheath was placed. A 5 Fr Glide Cobra catheter (Terumo, Somerset, NJ, USA) was advanced into the abdominal aorta, and the superior branch of the right renal artery was selected over a Glidewire (Terumo). The Glidewire was then exchanged for a J-wire. Over this wire-catheter combination, a 6 Fr, 45 cm Destination® sheath (Terumo, Somerset, NJ, USA) was advanced into the proximal right renal artery.

To facilitate branch protection and improve control, a Bard Aclipse® 6 mm × 20 mm dual-lumen balloon catheter (Becton Dickinson, Tempe, AZ, USA) was advanced over a 0.014 inch Chikai Black® microwire (Asahi Intecc, Aichi, Japan) into the distal branch of the superior right renal artery supplying the arteriovenous malformation. After balloon inflation, an occlusion arteriogram was performed to delineate the arterial-venous anatomy.

Concurrently, a 2.0 cm PX Slim microcatheter (Penumbra Inc., Alameda, CA, USA) and another Chikai microwire were advanced alongside the balloon catheter and navigated into the venous sac. Balloon occlusion venography confirmed optimal microcatheter positioning. Multiple Ruby® coils (Penumbra Inc., Alameda, CA, USA) ranging from 36 mm to 40 mm were deployed into the venous sac through the PX microcatheter under intermittent arteriographic guidance. A total of six coils were placed.

A repeat angiogram demonstrated markedly reduced flow through the arteriovenous malformation. With the balloon catheter reinflated, a final balloon-occluded arteriogram confirmed diminished filling. Onyx® 34 (Medtronic, Irvine, CA, USA) embolic agent was then administered through the PX microcatheter under balloon occlusion, delivering a total of 0.7 cc, with care taken to prevent off-target embolization.

**Figure 4:**
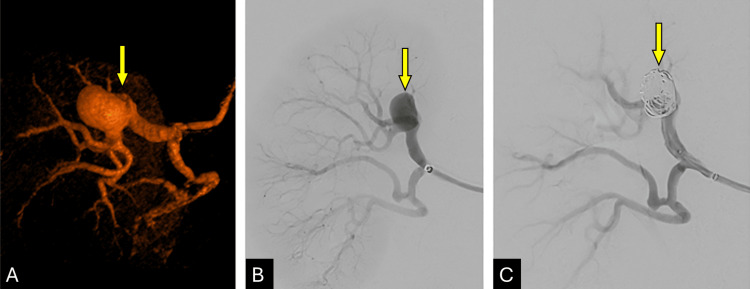
Treatment of a Right Renal Artery Aneurysm (A) 3D reconstruction showing FMD and aneurysm in the superior division of the right renal artery (yellow arrow). (B) Anteroposterior angiographic view of the saccular aneurysm (yellow arrow). (C) Subtracted image showing coil mass and deployed stent from the main to the superior division (yellow arrow).

Final angiography showed a well-expanded device with complete cessation of flow within the aneurysm sac and preserved perfusion to the inferior pole cortex (Figure 4). The patient tolerated the procedure without complications and was discharged within 24 hours. Follow-up imaging at six months confirmed complete aneurysm exclusion and stent patency.

The procedural durations and corresponding fluoroscopy times for each case are summarized in Table 2. 

**Table 2:** Procedural Duration and Fluoroscopy Time per Case

Case	Duration of Procedure (min)	Fluoroscopy Time (min)
One	134	28.1
Two	91	24
Three (a)	132	49.1
Three (b)	136	50.2

Postoperative management and follow-up

All patients were discharged within 24 hours, with no incidence of incision site infection, hematoma, bleeding, thrombosis, or coil and stent migration. Dual antiplatelet therapy with aspirin and clopidogrel was continued for three months post-procedure. Follow-up imaging at three to six months demonstrated persistent aneurysm occlusion, intact stent positioning, and preserved distal perfusion. There was no evidence of coil compaction, recanalization, or branch occlusion. None of the patients required additional interventions.

Results

Four wide-neck renal artery aneurysms were treated in three female patients, with one patient requiring bilateral intervention. The mean age was 62.3 years. All patients had both hyperlipidemia and elevated BMI, reflecting a shared cardiovascular risk profile. Two cases involved right renal artery aneurysms, and two involved the left. One aneurysm was located at a branch bifurcation. Although precise neck diameters were not consistently reported, all treated aneurysms met angiographic criteria for wide-neck morphology (neck diameter >4 mm or dome-to-neck ratio <1.2) based on procedural technique and intraoperative imaging.

In all cases, endovascular treatment was successful, with complete occlusion of the aneurysm and preserved parenchymal flow. No intraoperative or postoperative complications were reported. All patients were discharged within 24 hours of admission. Imaging follow-up at three to six months confirmed aneurysm occlusion. Parenchymal flow was preserved in all cases.

## Discussion

The endovascular management of renal artery aneurysms with wide necks or involving bifurcations remains technically challenging due to the risk of compromising renal perfusion with conventional coiling. While multiple endovascular techniques have been reported for managing renal artery aneurysms, many struggle to preserve branch vessel perfusion, leading to ischemia/infarction [14]. This challenge highlights the clinical value of our approach, which achieves complete aneurysm exclusion while maintaining flow to the parenchyma.

All three patients in this case series were female, and all showed imaging findings consistent with fibromuscular dysplasia, such as a “string of beads” appearance. These findings are in line with those from the U.S. Registry for fibromuscular dysplasia and the Assessment of Renal and Cervical Artery Dysplasia International Academy (ARCADIA) registry, which both indicate a high prevalence of multifocal fibromuscular dysplasia (FMD) characterized by alternating areas of stenosis and dilation, and a substantial female predominance among affected patients [15,16]. Our patients also shared cardiovascular risk factors, such as hyperlipidemia, elevated BMI, and, in one instance, a family history of aortic aneurysm. Patients' clinical presentations were diverse, with three presenting with abdominal or flank pain and one progressing to hematuria.

Patients with fibromuscular dysplasia can present with arterial stenosis, aneurysms, vessel tortuosity, a string-of-bead appearance, or dissections in medium-sized arteries. In a multicenter study performed by Olin et al., the renal artery was reported as the second most affected vessel in patients with FMD, following the carotid artery [17]. Although the pathophysiological mechanisms are not clearly understood, aneurysm formation has been recognized as a vascular manifestation of FMD. A strong association of FMD and aneurysm has been documented in multiple case series as well as in the United States Registry for Fibromuscular Dysplasia [11]. Notably, the registry also reported that 64.8% of patients with renal artery FMD that underwent neuroimaging had coexistent extracranial carotid or vertebral artery disease, denoting the systemic nature of FMD and its tendency to compromise multiple vascular territories [11].

Importantly, all of the cases presented wide-neck saccular aneurysms and two cases involving arterial bifurcations, morphologies/locations where traditional coil embolization carries a higher risk. In each case, our technique achieved aneurysm occlusion while preserving distal perfusion. There were no major periprocedural complications. One patient developed a small parenchymal infarct, which was identified on follow-up imaging but was clinically silent [14].

These results support previous reports that flow diverter stents promote the safe and effective treatment of complex visceral aneurysms by redirecting flow and enabling progressive thrombosis of the aneurysm while maintaining side branch patency [6,11]. Adjunctive coils were employed to stabilize devices and improve the exclusion of aneurysm sacs.

The mean age of our cohort was consistent with prior literature (approximately 61 years). All aneurysms were discovered incidentally during imaging for unrelated symptoms, suggesting that widespread CT availability has facilitated early identification [14]. Finally, all patients were discharged within 24 hours of their endovascular treatment, which suggests that same-day endovascular treatment of RAAs may be feasible in appropriately selected patients [18]. Limitations of this case series include the small sample size and short-term follow-up. Nevertheless, the uniform technical success within this series demonstrates the merits of a flow-diverter-assisted approach for the management of RAAs. Larger, multicenter case series or registries may help define optimal device selection and long-term outcomes.

## Conclusions

Flow-diverter stents, used with or without adjunctive coil embolization, offer a safe and effective treatment strategy for wide-neck renal artery aneurysms, including wide-necked aneurysms and lesions located at arterial branch points. These findings are limited to a small sample size of the study and a short-term follow-up. Further studies with extended follow-up are needed to validate these outcomes and inform treatment guidelines.
